# Resource productivity and environmental degradation in EU-27 countries: context of material footprint

**DOI:** 10.1007/s11356-023-26631-z

**Published:** 2023-03-29

**Authors:** Muhammad Mushafiq, Błażej Prusak

**Affiliations:** Faculty of Management and Economics, Gdansk University of Technology, Gdansk, Poland

**Keywords:** Resource efficiency, Climate change, Ecological damage, Environmental Kuznets curve, Environmental economics

## Abstract

This study explores the relationship between the resource productivity and environmental degradation in European Union-27 countries. This study tests this relationship in context of high, moderate, and low material footprint sub-samples; these samples are formed utilizing the expectation–maximization machine learning algorithm. Using the panel data set of EU-27 countries from 2000 to 2020, linear and non-linear autoregressive distributed lag (ARDL) are applied for the symmetric and asymmetric evidence and to test environmental Kuznets curve (EKC), linear ARDL with the quadratic function is included. Results of the symmetric relationship find evidence of resource productivity’s impact on the environmental degradation. In full sample of EU-27, both symmetric and asymmetric methods show that the short run and long run increase of resource productivity lower the environmental degradation. Only long run asymmetric relationship in high material footprint subsamples supports that the resource productivity controls environmental degradation. Results of moderate material footprint sub-sample are mixed. However, low material footprint countries show that resource productivity in long run controls the environmental degradation in symmetry and only positive resource controls productivity in short run in asymmetric relationship. The reason for mixed results is the quadratic nature of sub-samples. EKC hypothesis is validated in moderate and low material footprint sub-samples. This research has many policy implications.

## Introduction

During the last decade, the average temperature of Earth’s surface has risen by nearly a degree (Lindsey and Dahlman 2023; Global Climate Report 2021). Sea levels rise, snow and ice cover decrease dramatically, species go extinct, and other severe ecological crises occur simultaneously (WMO 2021). Emission of greenhouse gases, the most significant from which is carbon dioxide, is the principal driver of global warming (Chien et al. 2022). Fossil fuels, such as coal, oil, and natural gas, are the primary source of carbon emissions. Ever since industrialization, the Intergovernmental Panel on Climate Change (IPCC) estimates that approximately 500 billion tonnes of carbon have already been deposited into the atmosphere. The growing economy has resulted in a dramatic increase in the consumption of natural resources (Aziz et al. 2021; Gyamfi et al. 2022; Nathaniel et al. 2021; Opuala et al. 2022; Usman et al. 2022a, b), which in response have resulted in a lot of carbon emissions (Shiogama et al. 2019).

The use of natural resources is crucial to the manufacturing process (Horváth and Zeynalov 2016; Zahoor et al. 2022). In addition to production, the mining, refining, and eventual disposal of resources are major sources of wealth and employment in many nations. These actions also have some degree of environmental impact (Ahmad et al. 2019; Ahmad and Wu 2022; Ahmad and Zhao 2018). Natural resources are also a component of ecosystems which allows the circulation of services such as weather management, flood mitigation, wildlife ecosystems, utilities, and cultural attractions, which are essential for the development of intellectual, societal, and manufactured capital. There are far-reaching environmental, economic, and social ramifications of using natural resource-derived inputs in manufacturing and consumption which influence generations to come (Khan et al. 2021, 2022; OECD 2015; Sun et al. 2022a, b).

The economy cannot function without the natural resources provided by the ecosystem. However, the economy also has a negative effects on the environment through its byproducts such as waste and emissions. The manufacturing process is dependent on the utilization of natural resources, while simultaneously contributing to environmental stress (Aftab et al. 2022; Asiamah et al. 2022; Filimonova et al. 2020). There is a vicious cycle caused by the deterioration of environmental quality, which impacts not just the quality and quantity of accessible resources as well as the psychological and physical well-being of people (Davidson et al. 2021; OECD 2016).

Global and national policy elites’ focus has shifted from other challenges to global warming, resource scarcity, water and food shortages, and waste buildup as a consequence of the environmental repercussions of industrialization and urbanization. Integrating economic, environmental, and social considerations has gained traction among policymakers. As a result, there is a greater demand of data like resource productivity and material footprints, which complement economic accounts by providing a physical perspective on the economy and the sustainable development (Bringezu et al. 2016).

As the scarcity of the natural resources increases alongside the environmental degradation at its peak in the recent history, it has become essential to explore how utilization of the natural resources impacts the environmental productivity. The paper’s propose novel contribution is the introduction of the idea of material footprint as a vital aspect in comprehending the connection between resource production and environmental deterioration. The article presents a more thorough and nuanced view on the issue by including material footprint in the research, providing useful insights for governments, corporations, and individuals striving towards a more sustainable future. This approach represents a significant advancement in the existing literature and has the potential to drive significant improvements in resource productivity and environmental protection.

**Fig. 1:**
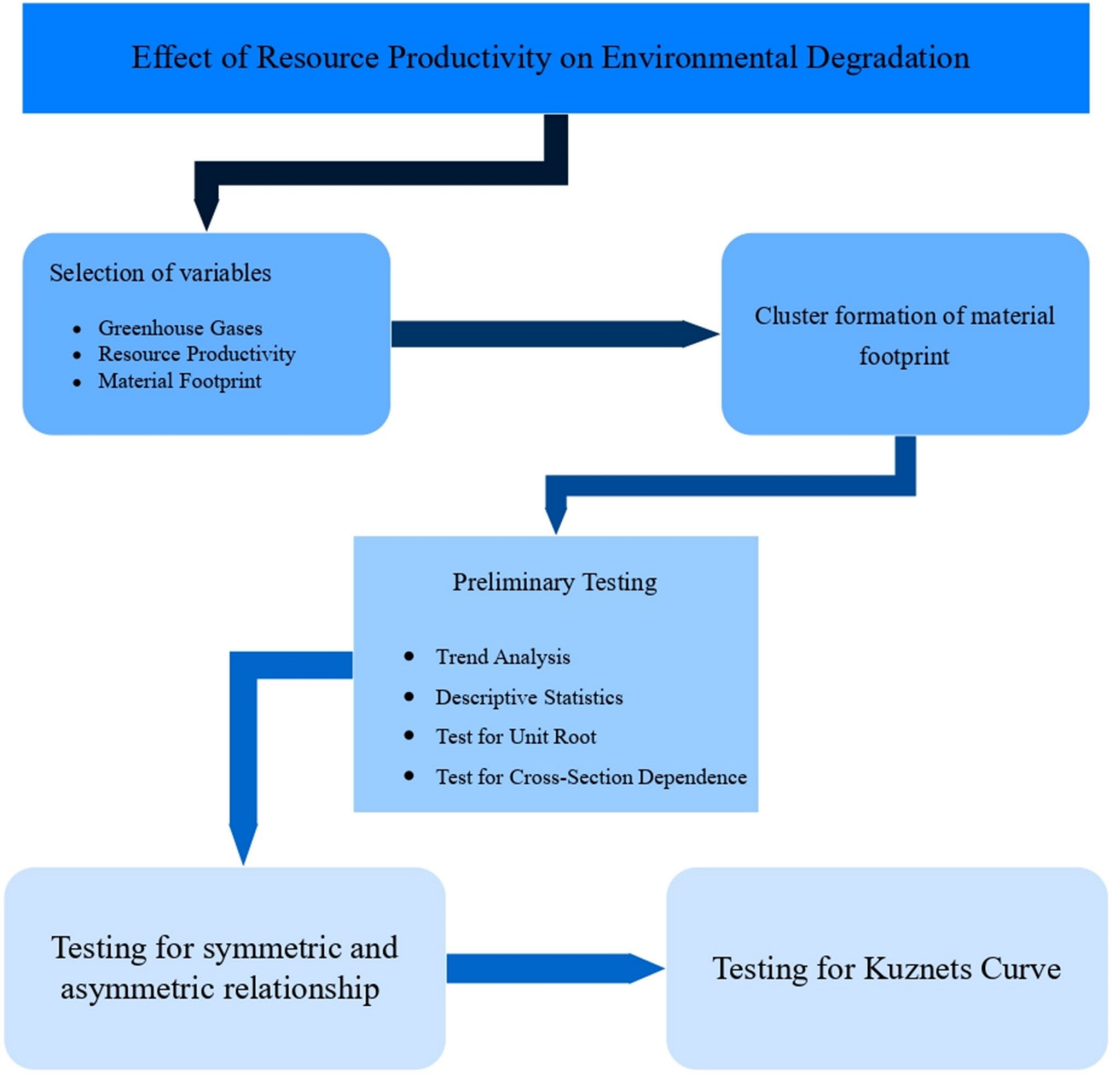
Scheme of the study. Source: own elaboration

Objective of the article is to find answer to the research question: can resource productivity control environmental degradation? If it does so, how is it different for those countries which have higher level of material footprint then those who have lower level of material footprint? The contributions of the study are as follows: (1) it conceptualizes that material footprint is essential to gauge the effect of resource productivity on environmental degradation, (2) this study forms clusters of countries with high, moderate, and low material footprint in EU-27, (3) the empirical evidence provided is for the long and short run, (4) the concept of asymmetry is tested, and (5) lastly, the modified EKC is tested. The results from the current study add substantial information to the prior literature. Figure 1 shows the framework of this study. “Conceptualization” section presents conceptualization. Methods and material succeeds the conceptualization. “Results and discussion” section presents the results, and “Conclusion and policy implications” section concludes the article.

## Conceptualization

Daly (1990) introduced the concept of sustainable development as a pragmatic approach, providing a theoretical explanation of it by modeling J.R. Hicks’ concept of income and arguing that the concept of income as the maximum amount that an individual or country could spend for a given time span and still be in the same financial position only at the end of the term has sustainability built into it. In addition, the theory posits that the resources, in both their supply and sink roles, must be utilized effectively and sustainably without driving them to extinction in the process, and the importance and scarcity would be hand-in-hand once nonrenewable resources become more scarce. It means that the pace of harvesting cannot be higher than the rate of regeneration, and that the rate of waste production cannot be higher than the rate at which the ecosystem can absorb it.

### Resource productivity

Recently, there has been numerous research on the natural resources in context of economic growth (Deng et al. 2022; Khan et al. 2022; Lee and He 2022; Tahir et al. 2022; Zeeshan et al. 2022) and energy (Liang et al. 2022; Sarwat et al. 2022; Zhang et al. 2022a, b). However, literature on resource productivity is extremely limited. The Organization for Economic Cooperation and Development (OECD) defines resource productivity as the efficiency with which an economy utilizes the materials that are derived from natural resources (physical inputs) to produce economic value (monetary outputs). OECD considers “resource productivity” in the context of wellbeing, which includes a qualitative component (e.g., the environmental impacts per unit of output produced with a given natural resource input).

This brief descriptions represent an ideal ecological and economic condition, but they do not take into account the interplay between the various goals that have been established. The worldwide extraction of resources and the processing of minerals, fuels, and food are responsible for almost half of the world’s greenhouse gas emissions, as well as more than 90% of water deficiency and the destruction of biodiversity (Sun et al. 2022a, b). Regardless of the efforts that EU companies have made over the last few decades to reduce their carbon footprint, 20% of the EU’s greenhouse gas emissions may be linked back to industry that still depends to roughly 90% on fresh materials mined from the earth (European Commission 2019).

Across the globe, governments are enacting new rules to improve resource productivity in order to keep up with the rising need for essentials like electricity, water, and materials. The Roadmap to a Resource Efficient Europe (RREE) and the European Green Deal (EGD) are two reference texts for resource productivity strategy in Europe. Even as Roadmap seeks to “increase economic performance while lowering strain on natural resources” (European Commission 2011), the Green Deal seeks to turn the EU into “a just and progressive society, with a modern, resource-efficient and productive economy that has no net emissions of greenhouse gases in 2050 and where economic development is decoupled from resource usage” (European Commission 2019).

In recent years, in the course of OECD work on material flows and resource productivity and on green growth, work by the UNEP International Resource Panel, and the public consultation process of the EU Resource Efficiency Roadmap, the necessity to apply more comprehensive indicators has been articulated by a large number of stakeholders, including policy makers, civil society, and academia. The main point of critique on the domestic material consumption (DMC) indicator is that countries can apparently reduce their national material consumption and improve their material productivity by dislocating material-intensive industries to other countries and substituting domestic extraction by imports (OECD 2008).

### Material footprint

The material footprint (MF) of a nation is the sum of its MFs for biomass, fossil fuels, metal ores, and non-metal ores, while the overall MF is the attribution of global material extraction to domestic ultimate demand of the country. Wiedmann et al. (2015) defined the term material footprint (MF) and described it as the worldwide allocation of utilized extracted raw materials to the end demand of an economy. They were influenced by the work of Galli et al. (2012), Moran et al. (2013), and Steen-Olsen et al. (2012). In contrast to measures of conventional economy-wide material flow accountancy, which are focused on apparent physical consumption (Fischer-Kowalski et al. 2011; Kovanda et al. 2012), the MF does not really document the actual physical movement of resources in and between nations; rather, it elaborates the connection between the initial stage of a production process (where natural resources are harvested from the natural environment) and its completion (in which the final product is consumed).

The material footprint of the European Union (EU) is the total quantity of raw materials needed to produce and deliver products and services to EU residents, both inside and outside the EU. The material footprint is a measurement of the total amount of resources needed to meet EU consumer demand, including those resources that must be harvested and imported from outside the EU. The material footprint tracks the use of natural assets by the economy after they have been extracted (European Environmental Agency 2022).

### Environmental aspect of resource productivity

A very limited research has been conducted on the aspect of resource productivity’s impact on the environmental quality. Adebayo et al. (2022) conducted a research that investigated at how natural resource depletion and globalization affect environmental quality in developing nations. The research uses empirical methods, including the fully modified ordinary least squares and the Method of Moments Quantile Regression, to determine that globalization moderates natural resources, hence enhancing environmental quality in the nations under consideration. On the other hand, the research has demonstrated the function of domestic material consumption in environmental quality, even when the moderating influence of globalization is ignored (Alola et al. 2021; Baniya and Aryal 2022; Usman et al. 2022a, b). Both Alola et al. (2021) and Usman et al. (2022a, b) used quantitative approaches that differed from one another to find that domestic material consumption degrades quality of the environment in a sample of 28 nations within the European Union. Using linear regression, Clodniţchi and Tudorache (2022) revealed a considerable negative relationship between EU-27 nations’ resource productivity and overall emissions of greenhouse gases. Alola and Adebayo (2022) found that in the Nordic nations, raw material productivity reduces greenhouse gas (GHG) emissions by employing symmetric and asymmetric autoregressive distributed lag (ARDL) model. Despite the preceding works, there remains a significant vacuum in literature of resource productivity, particularly as it relates to material footprints. Therefore, this study proposes that in setting of low material footprint, the impact of resource productivity on the environmental degradation should be higher as the overall consumption has been efficient. This can be explained as resource productivity when the country has higher level of material footprint, it is consuming more resources and this implies that the productivity is not at par it contributes towards the environmental degradation; however, when the country has lower level of material footprint, the consumption is somewhat efficient, and it eventually lowers the environmental degradation. The following hypothesis is presented from the above discussed literature.

- H1: Higher level of resource productivity lowers the environmental degradation in lower material footprint countries.

The study further proposes the idea that the relationship of resource productivity and environmental degradation is not linear. Initially, lower level of resource productivity increases the environmental degradation; however, when the eventually the resource productivity is increased to a certain level, it starts controlling the environmental degradation. However, this depends on the level of material footprint as well. In high material footprint countries, the high level of resource productivity might prompt over industrialization. To prove this idea, this study focuses on environmental Kuznets curve, originally proposed by Kuznets (1955) which explained that income disparity would increase at the outset of economic growth before leveling off. When economies expand, pollutant emissions rise, and environmental quality deteriorates at first. However, after incomes rise above a certain threshold (which will be different for different indicators), the tendency reverses, and economic growth ultimately improves environmental conditions (Shahbaz et al. 2013; Stern 2018). Application of EKC has been multifaceted (AlKhars et al. 2022), majorly focused on the nexus between energy, economy and environment (Akadırı et al. 2021; Arouri et al. 2012; Charfeddine 2017; Charfeddine and Ben Khediri 2016; Pata 2021) as well as on ecological footprint and carbon emissions (Ali et al. 2021; Altıntaş and Kassouri 2020; Ansari 2022; Mrabet and Alsamara 2017). According to the findings (AlKhars et al. 2022), the EKC hypothesis can only be proven correct in the long-term for the Gulf Cooperation Council (GCC) nations represented in the panel. In addition to this, they discovered that the pattern of economic production mirrors the pattern of economic freedom. Therefore, the EKC hypothesis is only verified in the long-term when economic freedom is utilized rather than economic development as the primary variable.

Findings of Arouri et al. (2012) demonstrated that estimated long-run coefficients of income and its square are consistent with the EKC hypothesis in the majority of the Middle East and North African (MENA) countries that were investigated. However, the turning points were found to be quite low in some cases and quite high in other cases, which resulted in weak evidence in favor of the EKC hypothesis. Even though there was overall economic development in the MENA area from 1981 to 2005, there was still a drop in the amount of CO2 emissions that were produced per person in that region.

Research of Charfeddine and Ben Khediri (2016) verified the existence of the environmental Kuznets curve (EKC). Furthermore, they found an inverted U-shaped link between economic growth and CO2 emissions in United Arab Emirates. Charfeddine (2017) discovered evidence to support the EKC hypothesis about the carbon ecological footprint and CO2 emissions proxies and the ecological footprint proxy had a U-shaped behavior. Pata (2021), while exploring EKC for ecological footprint, reported an inverted U-shaped EKC link between economic complexity and environmental pollution for the USA.

From the perspective of ecological footprint and carbon emissions (CO2), using CO2 emissions, the inverted U-shaped connection (EKC hypothesis) was found to be invalid in Europe by Altıntaş and Kassouri (2020). The EKC hypothesis is contingent on the selection of environmental indicators. Using CO2, CH4, and ecological footprint as environmental indicators, Ali et al. (2021)’s findings support the existence of inverted-U-shaped EKC across all OIC (Organization of Islamic Cooperation) country groupings. However, a U-shaped EKC appears in OIC nations as a whole and in OIC countries with lower incomes when N2O is employed. The results of Ansari (2022) confirmed the EKC theory for the environmental impact proxies. For the CO2 emission, the EKC hypothesis does not hold. Empirical evidence by Mrabet and Alsamara (2017) suggested that the inverted U-shaped hypothesis fails to hold when considering CO2 emissions in Qatar, but it does so when ecological footprint was considered. Applying this idea to the resource productivity, the following hypothesis is proposed.

- H2: The relationship between resource productivity and environmental degradation is in inverted U shape

## Methods and material

The research of the study is focused on the EU-27 countries due to the reason that almost 18% of the world greenhouse gases are from this region. Table 1 shows all the information regarding dataset used in the study. Environmental degradation is measured through the total greenhouse gases, whereas the resource productivity itself is a measure available at EuroStat. Unit of measure is tonnes per capita and euro per kilogram for greenhouse gases and resource productivity, respectively. Frequency of data is annual for 21 years from 2000 to 2020. This study uses material footprint as a classifier; the data for material footprint was obtained from United Nations Environmental Programme (UNEP). Data for material footprint is collected from year 1990 and 2016. The data of material footprint was not available for the Czech Republic.

**Table 1:** Dataset information

	Environmental degradation	Resource productivity	Material footprint
Proxy	Greenhouse gases	Resource productivity	Material footprint
Unit	Tonnes per capita	Euro per kilogram	Material footprint per capita
Time frequency	Annual	Annual	Annual
Time range	2000–2020	2000–2020	1990 and 2016
Region	EU-27	EU-27	EU-27*
Data source	EuroStat	EuroStat	UNEP

^*^Data for Czech Republic was not available. Source: own elaboration

**Fig. 2:**
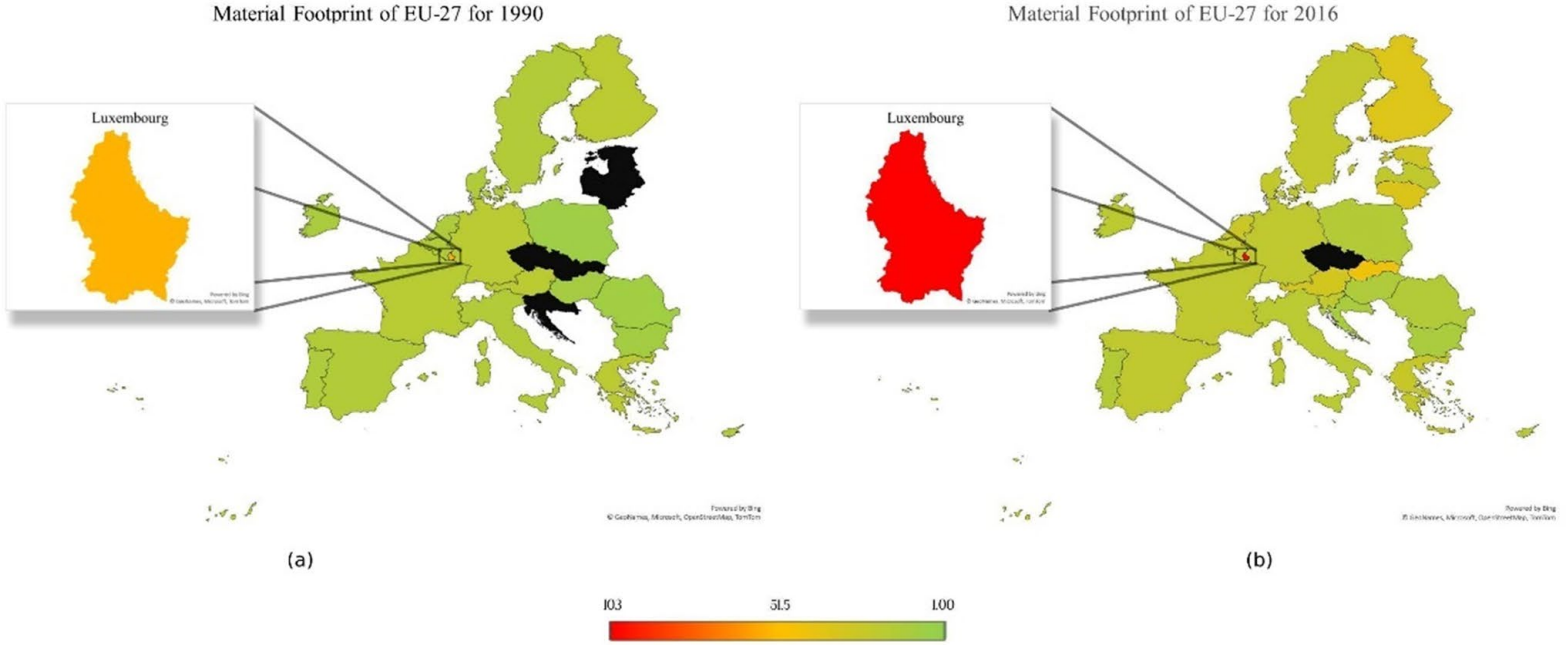
Mapping material footprint in EU-27 1990 (**a**) vs 2016 (**b**). Source: own elaboration based on publicly available data at EuroStat database

**Fig. 3:**
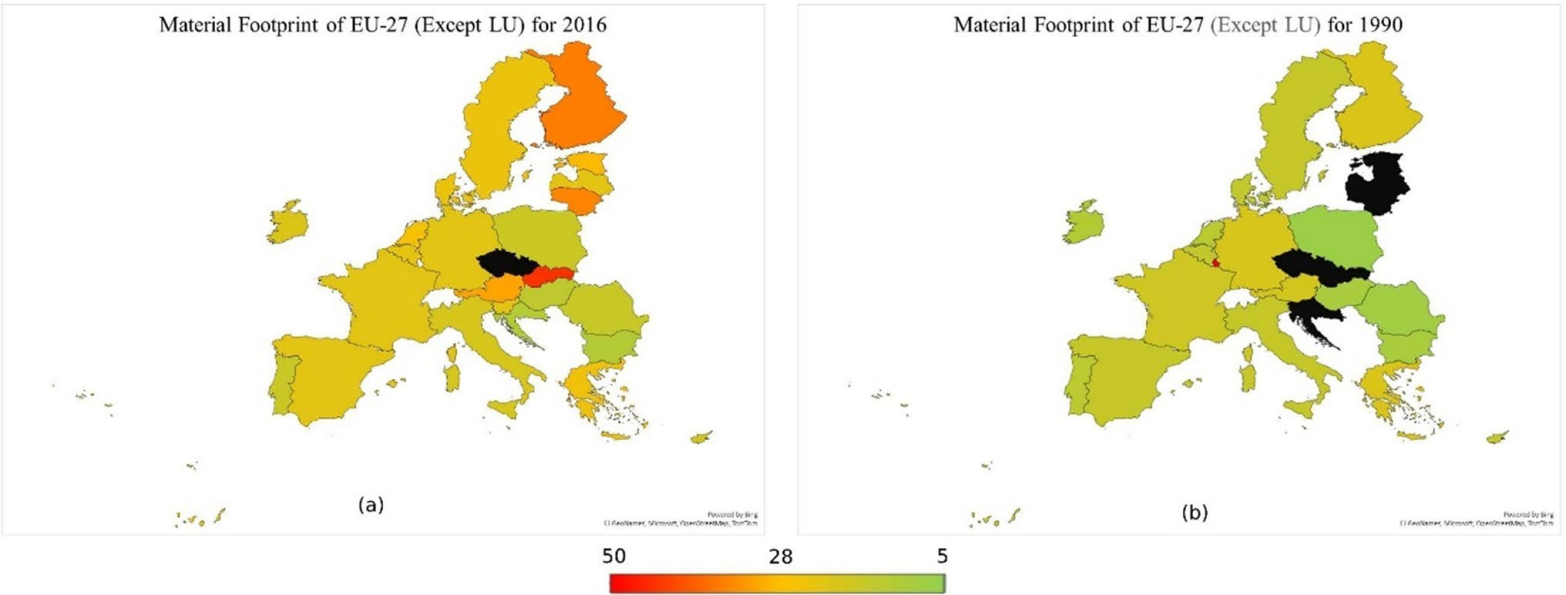
Mapping material footprint in EU-27 (except Luxembourg) 1990 (**a**) vs 2016 (**b**). Source: own elaboration based on publicly available data at EuroStat database

**Fig. 4:**
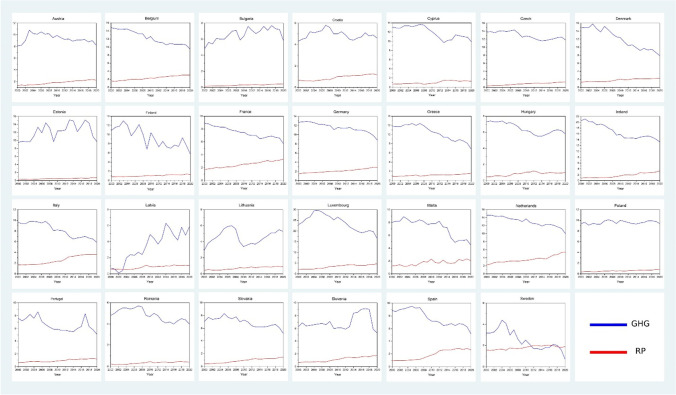
Trend of resource productivity and environmental degradation. Source: own elaboration based on publicly available data at EuroStat database

Figures 2, 3, and 4 show trend of variables and classifiers. Figure 1a and b show map of material footprint EU-27 in 1990 and 2016, respectively. The legend shows the range of material footprint, red being the highest material footprint country and green being the lowest, whereas black shows no availability of data. Luxembourg has the highest level of material footprint, creating an outlier that disrupts the trend and clustering process. Therefore, Luxembourg is excluded from the material footprint data, so the trend is visible more prominently. However, to evaluate the relationship between resource productivity and environmental degradation, Luxembourg would be considered part of the cluster with nearest score of material footprint. Figure 3a and b show map of material footprint EU-27 in 1990 and 2016 with exception of Luxembourg, respectively. The overall material footprint has increased quite a lot from 1990 to 2016. Slovakia, Finland, Lithuania, and Austria have higher level of material footprint as compared to the rest of region.

Figure 4 shows the trend of variables of interest. One of the observable things is that the resource productivity has increased over the period of time and the environmental degradation has lowered, subsequently. Except for the case of Latvia, the environmental degradation has increased over time. Luxembourg has the highest level of greenhouse gases but also the highest level of resource productivity. Followed by Luxembourg is Ireland with highest level of greenhouse gases. Sweden has the lowest level of greenhouse gases in the EU-27, post 2018 the resource productivity surpasses the greenhouse gases. Greece, Malta, Romania, Slovakia, and Slovenia have lower level of greenhouse gases. Bulgaria, Estonia, Lithuania, Poland, and Romania have less than 1 Euro per kg of resource productivity until 2020.

### Clustering material footprint

**Fig. 5:**
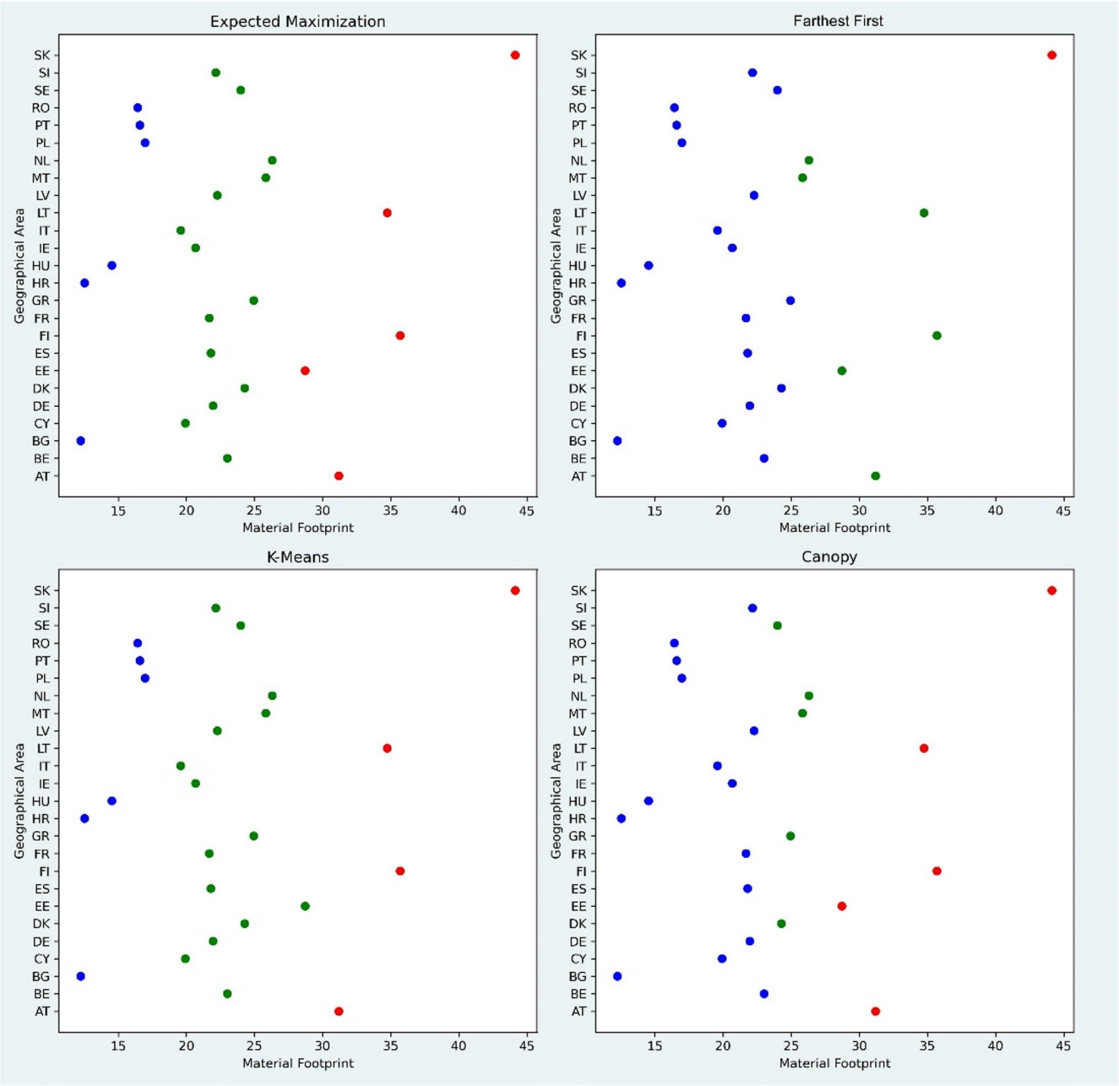
Clusters of material footprint. Source: own elaboration. Note: red cluster, high material footprint; green cluster, moderate material footprint; blue cluster, low material footprint

To test the relationship of resource productivity and environmental degradation at different levels of material footprint, it is necessary to form clusters at different level. This study using four machine learning clustering algorithms (1) expectation–maximization, (2) K-mean, (3) farthest first, and (4) canopy.^1^ All four of the methods were executed with pre-cluster-threshold of maximum number of clusters as 3. Figure 5 depicts results of each clustering method. The expectation–maximization cluster forms 3 clusters with 5 countries belonging to cluster 1, 14 to cluster 2, and 6 to cluster 3. The k-mean clustering shows similar results as compared to that of expectation maximization with 4 countries belonging to cluster 1, 15 to cluster 2, and 6 to cluster 3.

Farthest first and canopy provide different results, farthest first technique leads to only 1 member in cluster 1, 6 in 2nd and 18 in cluster 3. Canopy has 5 countries in clusters 1 and 2, and 15 in cluster 3, both farthest first and canopy are tail ended in formation of clusters. This study uses the clusters formed by expectation–maximization. Clusters 1, 2, and 3 can be named as high material footprint countries, moderate material footprint, and low material footprint countries. As discussed above, Luxembourg was excluded so that the cluster formation is free of any skewness; however, it will be added to nearest cluster. Therefore, it is included in cluster 1, i.e., high material footprint countries. Czech Republic is not included in any cluster but is part of complete EU-27 panel.

## Preliminary testing

### Descriptive statistics

Table 2 presents the descriptive statistics of variables from panel of EU-27 and all three clusters. The average greenhouse gas emissions of EU-27 from 2000 to 2020 are 9.243 tonnes per capita and the resource productivity is at 1.450 euro per kilogram. Countries with high material footprint have an average higher level of greenhouse gases (11.035 tonnes per capita) as compared to EU-27 as well as moderate and low material footprint. Resource productivity on average is 1.386 euros per kilogram in countries with high material footprint. Countries with moderate material footprint have higher productivity then the rest of sample, averaging at 1.801 euros per kilogram. Low material footprint countries have lower level of resource productivity averaging almost 64 cents per kilogram. High material footprint cluster has highest volatility in both greenhouse gases and resource productivity. Greenhouse gases and resource productivity both are positively skewed throughout the sample. Data is leptokurtic in EU-27 and high material footprint samples and for resource productivity in moderate material footprint sub sample, rest of the sub sample(s) have platykurtic distribution. Pairwise correlation between greenhouse gases and resource productivity is highest in the sub sample of high material footprint countries followed by full sample of EU-27 countries.

**Table 2:** Descriptive statistics

		EU-27		High MF		Moderate MF		Low MF	
	Description	GHG	RP	GHG	RP	GHG	RP	GHG	RP
Descriptive stats	Obs	567	567	126	126	294	294	126	126
	Mean	9.243	1.450	11.035	1.386	9.389	1.868	6.500	0.638
	Std. dev	4.641	0.966	6.520	1.078	4.067	0.878	1.716	0.318
	Variance	21.53	0.932	42.52	1.162	16.54	0.770	2.946	0.101
	Skewness	1.344	1.168	1.363	1.500	0.075	0.957	0.682	0.307
	Kurtosis	6.115	4.260	4.018	4.444	2.907	4.229	2.374	1.965
Percentiles	1%	1.600	0.169	3.400	0.285	0.600	0.550	4.000	0.148
	99%	26.60	4.379	29.50	4.379	20.20	4.793	10.00	1.256
Pairwise Corr	GHG	1.000	0.352***	1.000	0.672***	1.000	0.072	1.000	− 0.110
	RP	0.352***	1.000	0.672***	1.000	0.072	1.000	− 0.111	1.000

^***^, **, and * represent the level of significance at 1%, 5%, and 10%, respectively. Source: own elaboration

Results of unit root tests and cross-section dependence test are presented in Table 3. Greenhouse gases and resource productivity both are integrated at order one *[I (1)]*. Furthermore, the results of the Pesaran CD test indicate that the cross-sections of both variables are based on a heterogeneous structure. Because of this underlying heterogeneity and non-stationarity in the panel data series, the fundamental framework for estimation offered in this paper is suitable for the studies employed herein. In conclusion, the results of the unit root test provide validity to the panel-ARDL model’s status as the optimal estimation framework for the purpose of this study.

**Table 3:** Unit root test

	EU-27		High MF		Moderate MF		Low MF	
Description	GHG	RP	GHG	RP	GHG	RP	GHG	RP
Augmented Ducky Fuller I (0)	28.99	22.32	10.6	3.145	5.646	9.892	12.21	7.026
Augmented Ducky Fuller I (1)	201.7***	273.3***	60.35***	65.47***	87.07***	147.4***	46.12***	51.25***
Im-Pesaran-Shin I (0)	4.788	3.034	0.722***	1.817	6.308	2.686	− 0.433	0.689
Im-Pesaran-Shin I (1)	− 6.840***	− 10.69***	− 4.355***	− 5.302***	− 3.530***	− 7.871***	− 4.016***	− 4.528***
Perasan CADF I (0)	− 1.340	− 2.572***	− 1.072	− 2.340*	− 0.770	− 2.468***	− 1.239	− 2.406*
Perasan CADF I (1)	− 3.420***	− 3.323***	− 3.414***	− 3.925***	− 2.953***	− 3.244***	− 2.653**	− 3.377***
Perasan CD	34.31***	77.41***	5.706***	16.99***	23.79***	38.74***	4.104***	15.54***
No of cross-sections	27	27	27	27	27	27	27	27

^***^, **, and * represent the level of significance at 1%, 5%, and 10%, respectively. Source: own elaboration

### Linear ARDL

Since ARDL may be used with either an integrated order of zero (I(0)) or one (I(1)) (Akmal 2007)^2^ it was chosen. The methods used to assess the connection between the two are those proposed by Salisu and Isah (2017). To begin, it is believed that both gains and declines in resource productivity would have an equal impact on environmental deterioration. This assumption is then dropped so that the study may take into consideration both kinds of shifts. As a result, the model’s symmetrical panel ARDL may be expressed as:

1 $$\begin{array}{l}{\Delta g}_{it}= {\exists }_{0i}+{\exists }_{1i}{g}_{i, t-1}+{\exists }_{2i}{r}_{i, t-1} + \sum\limits _{j=1}^{N1} {\psi }_{1ij}{\Delta g}_{t-j }+ \sum\limits _{j=1}^{N2}{\psi }_{2ij}{\Delta r}_{t-j }+{\mu }_{i}+ {\varepsilon }_{it}\\ i=\mathrm{1,2},\dots ,N; t=\mathrm{1,2},\dots ,N\end{array}$$

Here, ${g}_{it}$ is the log of greenhouse gases emitted; ${r}_{it}$ is the log of resource productivity; ${\mu }_{i}$ is the group-specific effect; i is the country; and t is time period.

Assuming that ${\Delta r}_{t-j }=0$, the long run slope (elasticity) coefficient is calculated for each cross-section as $-\frac{{\exists }_{2i}}{{\exists }_{1i}}$. Accordingly, the short-term forecast for resource productivity is calculated to be ${\psi }_{ij}$. Equation (1) may be rewritten to incorporate an error correcting term in the following format:

2 $${\Delta g}_{it}= {\delta }_{i},{\upsilon }_{i,t-1}+\sum\limits_{j=1}^{N1}{\psi }_{1ij}{\Delta g}_{t-j }+\sum\limits_{j=1}^{N2}{\psi }_{2ij}{\Delta r}_{t-j }+{\mu }_{i}+ {\varepsilon }_{it}$$

where ${\upsilon }_{i,t-1}= {g}_{i,t-1}- {\phi }_{i}{r}_{t-1}$ is the linear error correction term for each unit; the parameter ${\delta }_{i}$ is the error-correcting speed of adjustment term for each unit which is also equivalent to ${\exists }_{1i}$. Using the formula $-\frac{{\exists }_{Ni}}{{\exists }_{1i}}$, we can get the values for each of the ${\psi }_{ni}$ parameter. The assumption of a symmetric influence of resource productivity on GHG is based on the fact that, as shown in Eqs. (1) and (2), resource productivity cannot be decomposed into positive and negative variations. To test the EKC, same procedure was followed as in Eq. (1) with addition of the squared value natural log of resource productivity in the equation. Equation (1) can be rewritten as:

3 $${\Delta g}_{it}= {\exists }_{0i}+{\exists }_{1i}{g}_{i, t-1}+{\exists }_{2i}{r}_{i, t-1} +{{\exists }_{3i}{r}_{i, t-1}}^{2} + \sum\limits _{j=1}^{N1} {\psi }_{1ij}{\Delta g}_{t-j }+ \sum\limits _{j=1}^{N2}{\psi }_{2ij}{\Delta r}_{t-j }+ {\sum\limits_{j=1}^{N3}{\partial }_{ij}{{\Delta r}_{t-j }}^{2}}+{\mu }_{i}+ {\varepsilon }_{it}$$

### Non-linear panel ARDL

By contrast to the symmetric scenario, the nonlinear panel ARDL provides for a nonlinear reaction of greenhouse emissions to resource productivity. According to this scenario, greenhouse gas emissions are not expected to react similarly to positive and negative shocks. As a result, the asymmetric version of Eq. (1) looks like this:

4 $${\Delta g}_{it}= {\exists }_{0i}+{\exists }_{1i}{g}_{i, t-1}+{\exists }_{2i}^{+}{r}_{t-1}^{+}+{\exists }_{2i}^{-}{r}_{t-1}^{-}+ \sum\limits _{j=1}^{N1} {\psi }_{1ij}{\Delta g}_{t-j} +\sum\limits _{j=1}^{N2}({{\psi }_{2ij}^{+}{r}_{t-1}^{+}+{\psi }_{2ij}^{-}{r}_{t-1}^{-})}+{\mu }_{i}+ {\varepsilon }_{it}$$

where (positive) and (negative) resource productivity shocks are denoted by ${r}_{t}^{+}$ and ${r}_{t}^{-}$, respectively. Long run (elasticity) coefficients ${r}_{t}^{+}$ and ${r}_{t}^{-}$ can be found by -$-\frac{{\exists }_{2i}^{+}}{{\exists }_{1i}}$ and $-\frac{{\exists }_{2i}^{-}}{{\exists }_{1i}}$, respectively. These disruptions are determined by positive and negative partial sum decompositions of resource production.

$$\begin{array}{l}{r}_{t}^{+}=\sum\limits _{k=1}^{t}{\Delta r}_{ik}^{+}=\sum\limits_{k=1}^{t}\mathrm{max}({\Delta p}_{ik},0)\\ {r}_{t}^{-}=\sum\limits_{k=1}^{t}{\Delta r}_{ik}^{-}=\sum\limits_{k=1}^{t}\mathrm{min}({\Delta p}_{ik},0)\end{array}$$

The error correction version of Eq. (4) yields the following:

5 $${\Delta g}_{it}= {\tau }_{i}{\xi }_{i,t-1}+ \sum\limits _{j=1}^{N1} {\psi }_{1ij}{\Delta g}_{t-j} +\sum\limits_{j=1}^{N2}({{\psi }_{ij}^{+}{r}_{t-1}^{+}+{\psi }_{ij}^{-}{r}_{t-1}^{-})}+{\mu }_{i}+ {\varepsilon }_{it}$$

In the asymmetric panel ARDL provided in Eq. (4), ${\tau }_{i}$ is the coefficient of error correction term that quantifies how long it takes the system to converge to its long run equilibrium in the presence of a shock, and the error-correction term ${\xi }_{i,t-1}$ captures the long run equilibrium.

## Results and discussion

First, we use the mean group technique and the pooled mean group method to estimate both models, and then we run these estimates through the Hausman test. When the null hypothesis is not rejected, the pooled mean group estimator is expected to be used, but the mean group estimator is assumed to be used when the null hypothesis is rejected. When the null hypothesis is being tested, the most effective estimator is the pooled mean group estimator, but when the alternative hypothesis is being tested, the mean group estimator is the most effective estimator. According to the results of the Hausman test, the pooled mean group estimator is the most effective estimator for simulating the connection between environmental degradation and resource productivity, with the exception of the symmetric ARDL in the high material footprint subsample.

Table 4 presents the results of symmetric impact of resource productivity on greenhouse gases in long and short run. In full sample of EU-27 both in long and short run, the higher resource productivity lowers the greenhouse gases. The magnitude is much higher in the long run. The results in sub samples differ a lot, in countries with high material footprint, resource productivity has no impact on the environmental degradation in long run and weak evidence of impact is found in short run that in higher material footprint countries, the resource productivity increases the environmental degradation. An explanation for this relationship can be that as the economy has achieved higher level of resource productivity, level of production increases as well, which in turn increases the emission of greenhouse gases and eventually contributing towards the environmental degradation. For countries with moderate material footprint, the resource productivity in longer run increases the environmental degradation; however, in short run, it contributes towards preservation of environment. Countries with low material footprint show a long run decline in environmental degradation due to increased resource productivity; however, no such effect is apparent for short run.

**Table 4:** Symmetric impact on greenhouse gases (*g)*

	EU-27		High MF		Moderate MF		Low MF	
	Coef	Std Err	Coef	Std Err	Coef	Std Err	Coef	Std Err
$r$	− 0.261***	0.062	− 1.078	0.339	0.759***	0.269	− 0.306***	0.084
$\Delta r$	− 0.050**	0.079	0.302*	0.160	− 0.280**	0.143	0.024	0.104
${\upsilon }_{i,t-1}$	− 0.416***	0.039	− 0.497***	0.125	− 0.185***	0.066	− 0.387***	0.030
Constant	0.904***	0.120	0..892***	0.333	0.343***	0.135	0.641***	0.074
Panel B								
PMG statistics								
No of observations	540		120		280		126	
No of cross sections	27		6		14		6	
Log likelihood	790.9		150.4		413.5		192.3	
Hausman test	1.250		− 29.67		0.820		2.030	
χ^2	0.263		0.000		0.365		0.154	

^***^, **, and * represent the level of significance at 1%, 5%, and 10%, respectively. Source: own elaboration

Table 5 presents the asymmetric evidence of resource productivity’s impact on the environmental degradation. Generally, the evidence for asymmetry is weak except only for full sample or low material footprint sub sample. For full EU-27 sample, the relationship between resources productivity decreases the environmental degradation in long run for positive resource productivity. In short run, positive resource productivity decreases environmental degradation and negative resource productivity increases the environmental degradation. Similar to full sample, in case of sub sample high material footprint, the positive resource productivity in long run decreases the environmental degradation; however, there is no significant effect in the short run. Moderate material footprint sub sample shows that increase in both positive and negative resource productivity decreases the environmental degradation similar to high material footprint the effect of resource productivity is not significant in short run. For the last sub sample of low material footprint, results of asymmetric positive effect in long run show that resource productivity increases the environmental degradation and in short run, the positive resource productivity decreases the environmental degradation. Table 6 presents the result of Wald test for existence of asymmetry. It reveals that there is not much of asymmetric effect when exploring the relationship between resource productivity and environmental degradation. Only full sample of EU-27 and sub sample of low material footprint provides evidence of asymmetry in short run.

**Table 5:** Asymmetric impact on greenhouse gases (*g*)

	EU-27		High MF		Moderate MF		Low MF	
	Coef	Std Err	Coef	Std Err	Coef	Std Err	Coef	Std Err
${r}^{+}$	− 0.276***	0.086	− 1.567**	0.743	− 0.295***	0.097	0.292**	0.133
${r}^{-}$	− 0.180	0.142	− 0.490	1.04	− 0.336***	0.165	0.041	0.141
${\Delta r}^{+}$	− 0.086*	0.049	− 0.164	0.189	− 0.012	0.057	− 0.194***	0.06
${\Delta r}^{-}$	0.247*	0.136	0.434	0.302	0.161	0.006	0.130	0.106
${\xi }_{t-1}$	− 0.397	0.039	− 0.226***	0.072	− 0.394**	0.057	− 0.440***	0.083
Constant	0.904***	0.110	0.514***	0.186	0.886***	0.162	0.828***	0.154
Panel B								
PMG statistics								
No of observations	540		126		280		120	
No of cross sections	27		6		14		6	
Log likelihood	804.8		131.6		418.6		194.5	
Hausman test	0.670		0.650		0.650		0.530	
${\chi }^{2}$	0.714		0.7218		0.7208		0.767	

^***^, **, and * represent the level of significance at 1%, 5%, and 10%, respectively. Source: own elaboration

**Table 6:** WALD test for verification of presence of asymmetry

		χ2	*p*
EU-27	$r$	0.29	0.5927
	$\Delta r$	4.63**	0.0314
High MF	$r$	0.80	0.3705
	$\Delta r$	2.30	0.1293
Moderate MF	$r$	0.04	0.8418
	$\Delta r$	0.53	0.4677
Low MF	$r$	1.17	0.2793
	$\Delta r$	6.55**	0.0105

^***^, **, and * represent the level of significance at 1%, 5%, and 10%, respectively. Source: own elaboration

**Fig. 6:**
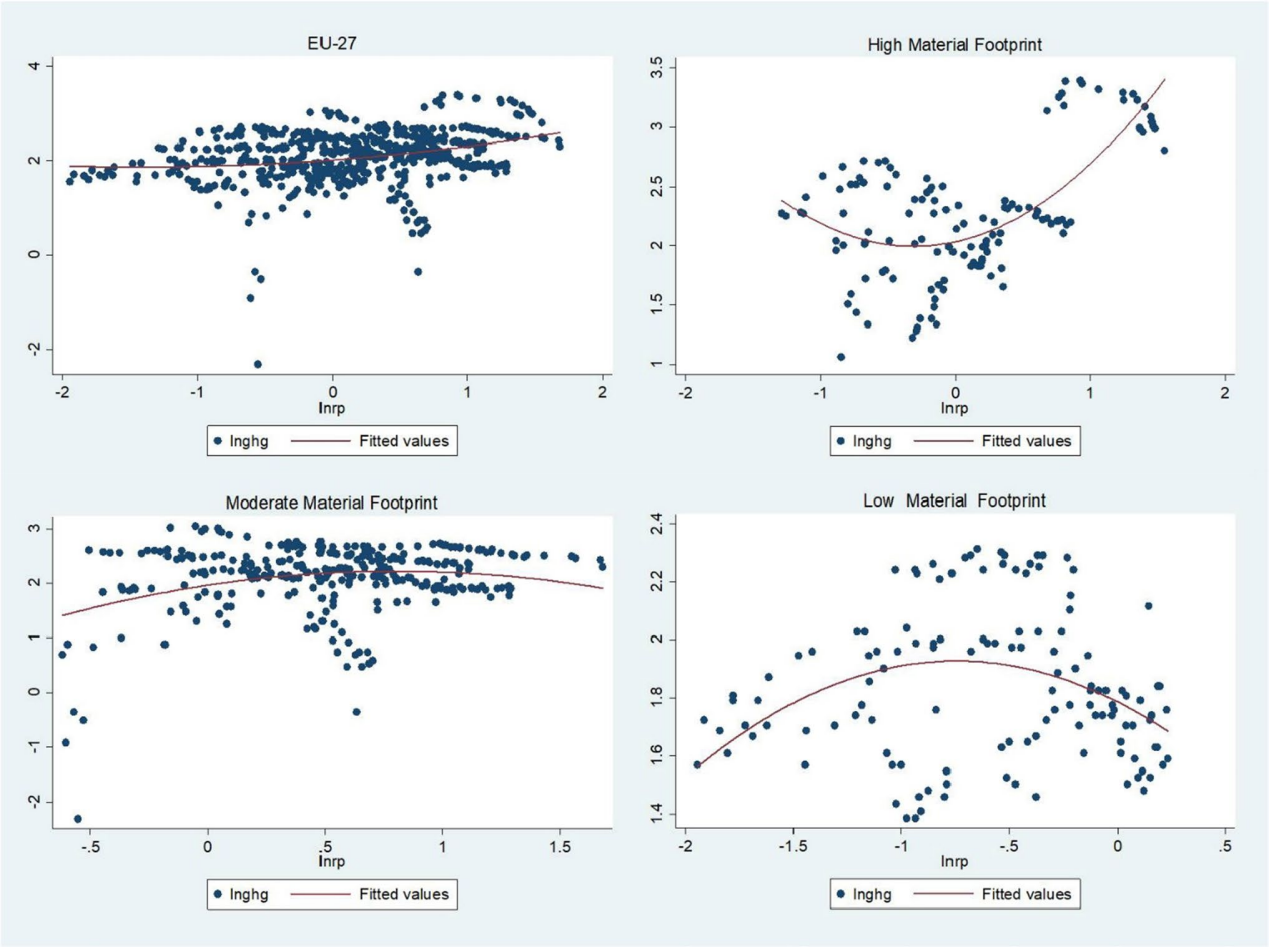
Quadratic fit plot. Source: own elaboration

Figure 6 shows the quadratic fitted plot of the greenhouse gases and resource productivity in EU-27, high material footprint, moderate material footprint, and low material footprint. This descriptive quadratic plot shows that the quadratic relationship is stronger and clearer in the high and low material footprint sub-samples.

**Fig. 7:**
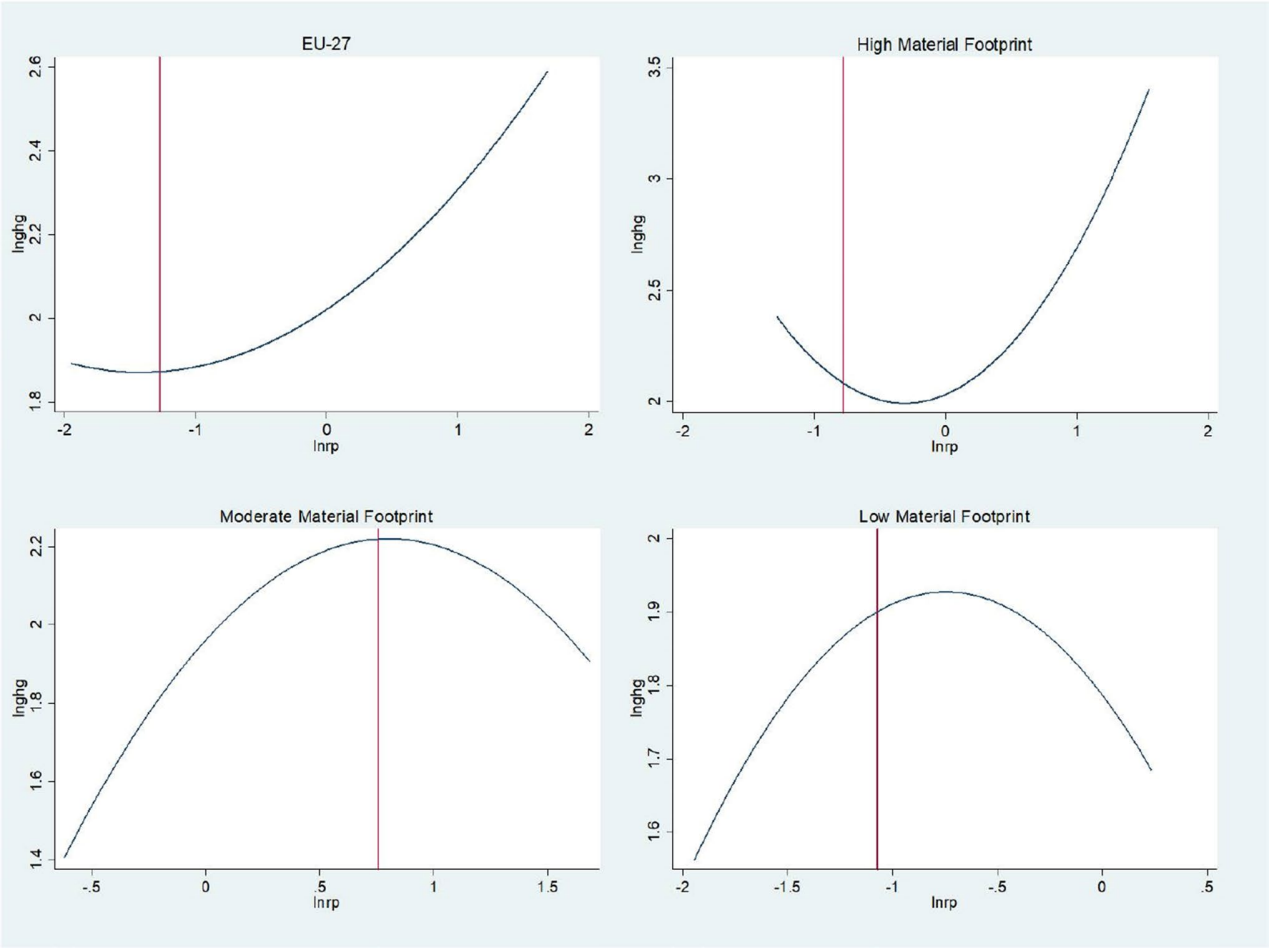
EKC graph with turning point. Source: own elaboration

Table 7 and Fig. 7 present the results of the testing for the EKC. The presence of the quadratic relationship is strong in the full sample and the two sub-samples, moderate and low material footprint countries. However, the quadratic impact of the resource productivity on environmental degradation is significant at 10%. Plotting the EKC in Fig. 7 shows that the proposed idea that resource productivity as a quadratic function of environmental degradation varies across the different levels of material footprint has been validated. As in the case of EU-27 and high material footprint sample, the EKC hypothesis that the resource productivity initially increases the environmental degradation and then lowers it has not been validated. The results of full sample are unclear, whereas in high material footprint countries, the EKC is U shaped, instead of inverted-U. This means that as the resource productivity increases, the high level of material footprint is utilized in more industrialized processes which in turn increases the environmental degradation. The EKC hypothesis is verified in the moderate and low material footprint countries. This implies that the resource productivity initially increases the environmental degradation, as the industrialization becomes more saturated. However, afterwards, the resource productivity lowers the environmental degradation in countries with moderate and low level of material footprint. The theoretical notion that a smaller material footprint is also necessary for a more significant influence of resource productivity on the deterioration of the environment. This concept is demonstrated to be accurate in this study for the long run by the fact that the long run coefficient of the symmetric panel ARDL for low material footprint countries has the largest contribution to reducing environmental degradation out of all the other significant coefficients. This finding validates the hypothesis for the long run. The results of this led to the approval of the first hypothesis and also confirmed that the link between resource productivity and environmental deterioration varies across different degrees of material footprint. These results are in line with the findings of two of the most prominently research carried out earlier (Alola and Adebayo 2022; Clodniţchi and Tudorache 2022).

**Table 7:** Testing for Kuznets curve

	EU-27		High MF		Moderate MF		Low MF	
	Coef	Std Err	Coef	Std Err	Coef	Std Err	Coef	Std Err
$r$	− 0.565***	0.071	− 1.094	1.029	0.374*	0.204	− 0.511***	0.111
${r}^{2}$	− 0.222***	0.04	− 0.705*	0.396	− 0.247***	0.079	− 0.239***	0.064
$\Delta r$	− 0.017	0.241	0.176	0.319	0.151	0.507	0.139	0.099
$\Delta {r}^{2}$	− 0.193	0.31	0.770*	0.412	− 0.854	0.591	− 0.121	0.231
${\upsilon }_{i,t-1}$	− 0.381***	0.051	0.025***	0.009	− 0.436***	0.068	− 0.507***	0.067
Constant	0.782***	0.151	1.039**	0.449	0.915***	0.178	0.858***	0.129
Panel B								
PMG statistics								
No. of observations	540		120		280		120	
No. of cross sections	27		6		14		6	
Log likelihood	820.8		153.7		433.8		198.4	
Hausman test	1.780		6.600		1.100		1.070	
${\chi }^{2}$	0.410		0.037		0.578		0.586	
Turning point	− 1.272		− 0.776		0.757		− 1.071	

^***^, **, and * represent the level of significance at 1%, 5%, and 10%, respectively. Source: own elaboration

In addition, the findings indicate that the second hypothesis of the study may be accepted since the EKC hypothesis was proven in the moderate and low material footprint samples. In these sub samples, the resource productivity gradually decreases the environmental degradation. The U-shaped relationship between resource productivity and environmental degradation, as seen in high material footprint countries, suggests that as resource productivity increases, environmental degradation also increases, despite efforts to improve resource efficiency. This outcome is often referred to as “strangling with over-industrialization.”

Based on the results, material footprint should be minimized wherever possible since this helps to maximize the use of available resources and minimizes the amount of waste produced. This, in turn, may have a substantial positive impact on an organization’s bottom line. In addition, lowering the material footprint may help an organization enhance its sustainability profile, which is something that is becoming an increasingly essential factor in the contemporary commercial environment. Companies that are viewed as being environmentally responsible tend to be more appealing to consumers and investors, which may have a favorable influence on the companies’ reputations as well as their bottom lines.

Material footprint is an essential tool for businesses that want to increase their resource productivity while simultaneously cutting waste and lowering their negative impact on the environment. When companies have a thorough awareness of their material footprint, they are better able to pinpoint areas in need of improvement, enhance their resource use, and generate economic benefits. This underlines how important it is to take into consideration the material footprint as part of an all-encompassing approach to the management of sustainable resources.

## Conclusion and policy implications

This study focusing on the resource productivity and environmental degradation from a context of material footprint using the symmetric method of evaluating the relationship found substantial evidence that resource productivity decreases the environmental degradation. Asymmetric evidence is relevant from the context of EU-27 but in the sub-samples formed from the material footprint. The theoretical idea that lower material footprint is also essential for stronger impact of resource productivity on the environmental degradation. This idea is validated in this paper for long run, as the long run coefficient of the symmetric panel ARDL for low material footprint countries has the largest contribution to lower the environmental degradation among all the other significant coefficients. Leading to acceptance of first hypothesis and also confirming the variation of the relationship between resource productivity and environmental degradation in different material footprint levels, these results are consistent with previous work. Moreover, the results show that the second hypothesis of the study is accepted as EKC hypothesis is validated in the moderate and low material footprint samples in these sub samples, the resource productivity eventually lowers the environmental degradation.

Going green till 2050 is a challenge at hand for the European Union countries as the sustainable development defined by Daly (1990) should be in terms of preserving the environment but also preservation of the resources is to be considered. Increasing resource productivity to lower the environmental degradation would lose all its purpose if only resource productivity remains in focus and not material footprint. There are three prime policy implications of this study. Firstly, the usage of fossil fuels is one of the largest contributors to the material footprint and transitioning to renewable energy sources can play a significant role in reducing it. The policy implications of this transition are numerous, as lower resource consumption and reduced environmental degradation can have far-reaching benefits.

From a resource productivity perspective, the use of renewable energy sources can increase efficiency and reduce waste, as they are often produced with fewer inputs and emissions than fossil fuels. This reduction in resource consumption can have a direct impact on the material footprint of a country, reducing its impact on the environment and improving resource productivity. In addition, the transition to renewable energy can also have indirect benefits for resource productivity. For example, it can reduce dependence on limited resources such as oil, which can become scarce over time, leading to price volatility and resource depletion. This transition can also promote innovation in clean energy technologies and help to create new jobs in the clean energy sector.

Secondly, as the results of testing EKC show in high material footprint countries, the EKC is U-shaped which can be interpreted as the resource productivity increases environmental degradation, one of the possible explanations for this is that as the resource productivity increases the countries instead of cutting the usage of resources, they strangle themselves with over-industrialization and creating EKC hypothesis suggests that economic development and resource productivity can have a complex relationship with environmental degradation. According to the EKC hypothesis, resource productivity initially increases environmental degradation, but as the level of development increases, resource productivity and environmental degradation become decoupled, and environmental degradation begins to decline. From a policy perspective, this result highlights the need to consider both the level of resource productivity and the context in which it is being applied when designing policies to reduce environmental degradation. In high material footprint countries, a more nuanced approach may be needed to ensure that increases in resource productivity do not lead to increased environmental degradation. Additionally, policies can be designed to incentivize companies to adopt more sustainable practices, such as reducing waste and emissions, and promoting resource efficiency and increase in environmental degradation.

Thirdly, considering this study, from the concept of the circular economy, it has the potential to significantly improve resource productivity, reduce waste, and lower environmental degradation. This is achieved by ensuring that resources are utilized more efficiently, reducing waste, and improving resource efficiency. However, as Daly (1990) highlights, it is important to manage resource extraction in a sustainable manner. From a policy perspective, this means that policymakers in the circular economy should focus on limiting resource extraction, so that it is done in a sustainable manner. This can be achieved through a range of measures, such as promoting the development of alternative technologies, encouraging resource recycling, and reducing dependence on finite resources. Additionally, policymakers can encourage the use of renewable resources, such as solar and wind power, to reduce reliance on non-renewable resources and lower the material footprint.

## Data Availability

Available on request.

## Footnotes

1 To read more about the clustering methods, kindly see the following literature (Do and Batzoglou 2008; Hochbaum and Shmoys 1985; Likas et al. 2003; MacQueen 1967; Mccallum et al. 2000).

2 ARDL would yield strong findings since several of the variables in this study are integrated at *I(1).*
